# Impact of Environmental Stresses on the Antibacterial Activity of Graphene Oxide (GO) Nanoparticles against *P. putida* Biofilms

**DOI:** 10.3390/microorganisms11030609

**Published:** 2023-02-28

**Authors:** Hussam Fallatah, Tim Overton, Hanene Ali-Boucetta, Konstantinos Gkatzionis

**Affiliations:** 1 Waste Management and Recycling Institute, King Abdulaziz City for Science and Technology (KACST), Riyadh 12354, Saudi Arabia; 2 School of Chemical, Engineering, University of Birmingham, Birmingham B15 2TT, UK; 3 Nanomedicine, Drug Delivery & Nanotoxicology (NDDN) Laboratory, School of Pharmacy, University of Birmingham, Birmingham B15 2TT, UK; 4 Department of Food Science and Nutrition, University of the Aegean, Metropolite Ioakeim 2, 81400 Myrina, Lemnos, Greece

**Keywords:** graphene oxide, biofilm, *P. putida*, environmental stresses, toxicity

## Abstract

As the production of graphene-based nanomaterials such as GO is increasing, it is expected that a large amount of GO waste will be generated. The environment (i.e., soil and aquatic systems) will be amongst the final repositories of these wastes which means important natural microbial communities in such environments will be at risk of GO exposure. However, little is known about how these communities respond to environmental stresses in synergy with the presence of GO. In this study, the effect of three different stress conditions: temperature (5, 25 and 40 °C); pH (5 to 9) and osmotic stress (51, 219 and 320 mM NaCl) in addition to GO treatment was investigated on the viability and physiology of biofilms and planktonic cells of soil bacterium *P. putida*. It was found that planktonic cells were more resistant to GO alone compared to biofilms. However, the cells were sensitive to GO when exposed to pH or osmotic stresses. Temperature was not found to influence the survival of biofilm with or without exposure to GO. However, low pH caused a reduction in colony-forming units (CFU) at pHs 5 and 6 for the pre-treated samples, while biofilms at pH 7–9 did not show any decrease. Interestingly, the post-treatment of planktonic cells or biofilms with GO showed a significant reduction in CFU at all pH ranges. The effect of higher osmotic stress in combination with GO resulted in a significant reduction in biofilms. These results show that the effect of stresses naturally occurring in the environment can be affected and changed when in combination with GO and can potentially affect the balance of natural biofilms.

## 1. Introduction

The increase in GO applications will increase the possibility of its discharge into the environment [1] and soil ecosystems are more likely to be a large recipient of nano-material contamination when compared with water and air [1]. Bacterial biofilms are widely distributed in soil and play a major role in nutrient recycling such as carbon, nitrogen, phosphorus, and sulfur. They are also frequently exposed to environmental changes such as temperature, pH and osmotic changes [2]. Generally, bacteria have the ability to grow at a wide range of environmental changes far from the optimal conditions and have the ability to adapt to these changes in order to survive and grow [3].

Bacteria in biofilms are more resistant to extreme environmental conditions or antimicrobial agents than those grown under planktonic conditions, making their more extracellular polymeric substances robust and difficult to destroy [4]. Since bacterial cells are embedded in extracellular polymeric substances (EPS), this may limit the transport of antibacterial agents (e.g., antibiotics) to cells [5].

The amount of EPS increases with high stresses which makes biofilms even more resistant. Lin et al. studied the EPS production of biofilm in response to temperature, pH and osmotic stresses in *P. putida* CZ1 [6]. In addition, the amount and compaction of EPS increased during specific conditions (low pH, high temperature and certain osmotic stresses), and showed that the EPS production is almost linearly and positively correlated with culture time, while the number of cells decreased to cope with nutrient depletion during aging [6]. Moreover, the study showed that EPS production formed a more hydrated microenvironment around cells, therefore increasing their tolerance.

Others stated that combination treatment such as that of increased temperature and antibiotics (ciprofloxacin, tobramycin, and erythromycin) shows synergistic effects against *Pseudomonas aeruginosa* biofilms [4]. It has been proposed that the transport of antibiotics through the EPS increases with increasing the temperature, suggesting synergistic effects on *P. aeruginosa* biofilms [4]. In addition, when the osmotic stress of the surrounding cells increased, the tolerance of cells to high hydrostatic pressure decreased [7]. 

Many studies investigated the effects of environmental stresses on biofilms during its developing stages [8,9] however only few investigated their impact on mature biofilms [10]. In addition, GO has been shown to have a significant effect against mature biofilm but not planktonic cells [11]. However, no study to-date has investigated the combined effects of environmental stresses and GO on mature biofilms. It is known that GO is a two-dimensional (2D) honeycomb with abundant functional groups on the surface and edges such as epoxy, carboxyl, and hydroxyl groups. These groups make GO hydrophilic and well dispersed in water. In the environment, GO could easily get re-dispersed in water when rain falls on soil contaminated with GO. 

One objective of our study is to determine the impact of the environmental stresses such as temperature (5, 25 and 40 °C), pH (5, 6, 7, 8 and 9) and NaCl (51, 219 and 320 mM) on 48 h *P. putida* biofilm and compare it to their corresponding planktonic cells. The aim of the study is to determine the impact of the environmental stresses on biofilm or planktonic cells and to assess the effects of GO on biofilms and planktonic cells after exposure to these environmental stresses.

## 2. Materials and Methods

### 2.1. Preparation of Graphene Oxide and Buffers

*Graphene oxide:* Graphite was purchased from Sigma Aldrich and GO preparation was as described in [11,12]. In brief, 0.2 g of graphite and 0.1 g of sodium nitrate (NaNO_3_) were combined with 96% sulfuric acid (H_2_SO_4_) in an ice bath and stirred for 20 min. Potassium permanganate (KMnO_4_, 0.6 g) was added to the mixture and stirred for 30 min. An amount of 9.2 mL of deionized (DI) water was slowly added followed by 3 mL of hydrogen peroxide (H_2_O_2_). Several washes were then carried out through centrifugation with deionized water; the sedimented GO was dried in an oven at 40 °C for 48 h and resuspended in DI. GO was stored in 4 °C until further experiments.

Citrate–phosphate Buffers: These buffers were used to obtain acidic buffers. Three pH values were used (5.0, 6.0 and 7.0) by mixing 0.1 M citric acid and 0.2 M dibasic sodium phosphate in the proportions indicated in Table 1 and adjusting the final volume to 100 mL with deionized water.

Tris-HCl Buffers: These buffers were used to obtain alkaline buffers at pH values of 7.0, 8.0 and 9 by mixing 0.1 M Tris(hydroxymethyl)aminomethane and 0.1 M hydrochloric acid (HCl). To achieve these pH values, 50 mL of Tris(hydroxymethyl)aminomethane was used and known volumes of HCl as described in Table 2 were mixed and adjusted to the final volume of 200 mL with deionized water. A pH meter (Beckman) was used to adjust the final pH.

*NaCl solutions* were used to stimulate osmotic stress by preparing 51, 219 and 320 mM (equivalent to 0.3, 1.3 and 1.9%) NaCl. An amount of 58.44 g of NaCl was initially dissolved in a final volume of 1 liter to make a 1 M solution. Then, dilutions were made to obtain these concentrations. 

### 2.2. Bacterial Strain, Culture Conditions and Biofilm Cultivation

The bacterial strain used for this study was *Pseudomonas putida* KT2440 and was maintained on tryptic soy agar (TSA) and incubated at 30 °C overnight then transferred to 100 mL of TSB and incubated at 30 °C for 24 h at 150 rpm. Cells were washed in phosphate-buffered saline followed by their inoculation in a CDC bioreactor as described in [11].

### 2.3. Environmental Stress and Quantification of Biofilm Incubated with GO

To study the effect of temperature, biofilms developed within the coupons in the CDC reactor were collected after 48 h. Coupons carrying biofilm bacteria onto them were incubated at 5, 25 or 40 °C with sterile deionized water (control) or 4 mL of GO at 85 µg/mL for 24 h with shaking at 80 rpm. The viability was tested by plate counting as described in [11]. For planktonic cells, CFU was taken after incubation with water or GO at the same temperatures and for the same times. One colony was transferred from a plate agar into 100 mL of TSB and incubated overnight. We added 10^8^ CFU/mL from the culture to 4 mL of H_2_O or 85 µg/mL GO for 24 h with shaking at 80 rpm and incubated at the same temperatures. CFUs were enumerated after incubations.

To study the effects of pH and osmotic stress, biofilms were grown in the CDC reactor for 48 h and were rinsed with PBS (pH 7.0) to remove non-adherent cells followed by adaptation at different pHs (5, 6, 7, 8 and 9) or for different osmotic stresses with NaCl (51, 219 and 320 mM) for 3 h at room temperature. For the pH experiment, two groups were used: the acid group included pH 5, 6, and 7 using citrate–phosphate buffers while the alkaline group included pH 7, 8 and 9 using tris-HCl buffers. 

Six coupons were used for each pH in this experiment: two coupons were exposed to different pH stresses without GO for 3 h and called pre-treatment (Figure S1). The other four coupons were also exposed to the same stresses for 3 h then incubated with either deionized water (control) or 85 µg/mL GO for 24 h at 25 °C with shaking at 80 rpm and called post-treatment. In addition, planktonic cells were treated similarly to biofilms. One colony was transferred from the agar plate into 100 mL of TSB and incubated overnight. We added 10^8^ CFU/mL from the culture to 4 mL of the different pHs (5, 6, 7, 8 and 9) or (51, 219 and 320 mM) NaCl. However, the free cells were washed with PBS two times after the incubation with pH or osmotic stress solutions, then incubated with either deionized water or 85 µg/mL GO. 

### 2.4. Confocal Microscopy

Biofilms were observed using Leica SPE-II confocal microscopy (Leica) by washing the coupons with PBS and staining the cells with SYTO9 (100 µM) and PI (100 µM) with 5 min incubation in the dark. Biofilms were observed using Leica SPE-II confocal microscopy (Leica) under 60× magnification and the images were obtained with a Leica DFC500 camera and Leica LAS AF software. 

### 2.5. Statistical Analysis

The statistical analysis was conducted using Student’s *t*-test to compare two means or one way analysis of variance (ANOVA) and the Tukey’s HSD post hoc test to compare several means were used for checking whether there was significant difference among samples using IBM SPSS Statistics software version 23. Differences were considered significant at *p* < 0.05.

## 3. Results

### 3.1. Effect of Temperature and GO on the Viability of Planktonic and Biofilm Cells

**Figure 1:**
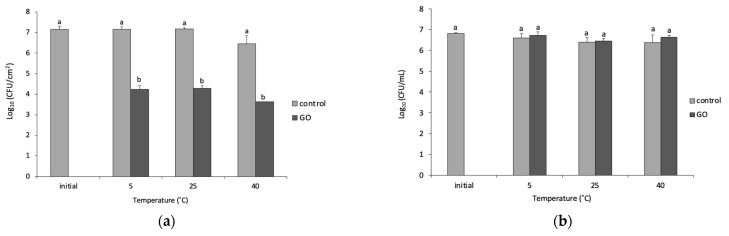
The viability of 48 h *P. putida* with and without GO incubated at 5, 25 and 40 °C for 24 h (**a**) biofilm and (**b**) planktonic cells. Initial indicates the viability of *P. putida* after sampling from CDC bioreactor. Mean values with different letters are significantly different (*p* < 0.05). Similar letters are non-significantly different, while different letters are significantly different. The data were analyzed with one-way ANOVA.

The chemical characterization of the obtained GO was described by Fallatah et al. [11]. Briefly, the GO was shown to have a diameter of >200 nm, a thickness of 1.8 ± 2 nm and a C:O ratio of 2:1 [11]. The effect of temperature on the viability of 48 h biofilms and planktonic cells was monitored by plate counting (CFU/m^2^) for biofilms and (CFU/mL) for planktonic cells. The controls in biofilms were not significantly affected at low or high temperatures as seen in Figure 1a. In addition, when comparing the biofilms exposed to 85 μg/mL GO for 24 h, different temperatures did not show any effects on the cell viability as shown in Figure 1a. However, when the GO-exposed biofilms were compared to their control counterparts, the growth showed a ~3-log reduction at 5, 25 and 40 °C. In addition, around a 3.5-log reduction in the number of CFUs was observed at 40 °C compared with the initial biofilm and ~3-log reduction was seen in comparison with the control biofilm at the same temperature.

As seen with biofilms, planktonic cells showed no reduction in CFU number for controls at all temperatures 5 °C, 25 °C and 40 °C as shown in Figure 1b. Moreover, there was no significant effect on planktonic cells when cells were exposed to 85 µg/mL GO at all temperatures or compared with the other controls. 

### 3.2. The Stability of GO in pH and NaCl Solutions

To assess the effect of environmental stresses such as pH and NaCl on bacterial cells after exposure to GO, it is crucial to investigate the stability of GO at the different pHs and NaCl concentrations. GO at 85 μg/mL was added to different pH buffers and solutions containing NaCl of similar concentrations used for the environmental stress and incubated for 24 h to identify the stability of GO in these solutions. Figure S2 shows the aggregation of GO at pH 5 and at 320 mM NaCl after incubation at room temperature. In the acidic buffer (pH 5), GO becomes protonated and hydrophobic; thus, it aggregates, and the dispersion becomes more transparent, while in the alkaline buffer (pH 9), GO becomes deprotonated and remains hydrophilic and, therefore, remains homogeneous. These results suggest that GO is not stable at any pH or NaCl concentration used in this study and, therefore, the bacterial cells would be treated with GO in deionized water after they have been exposed to environmental stresses.

### 3.3. Effect of pH and GO on the Cell Viability of Biofilms

To study the combined effect of pH and GO on biofilms, *P. putida* mature biofilms were incubated for 3 h at different pH values (5, 6, 7, 8 and 9) and then further incubated for 24 h with either H_2_O (control) or 85 µg/mL GO. An additional control was included in the study named pre-treatment. This was needed to investigate the effects of pH alone on the biofilms without the addition of GO.

**Figure 2:**
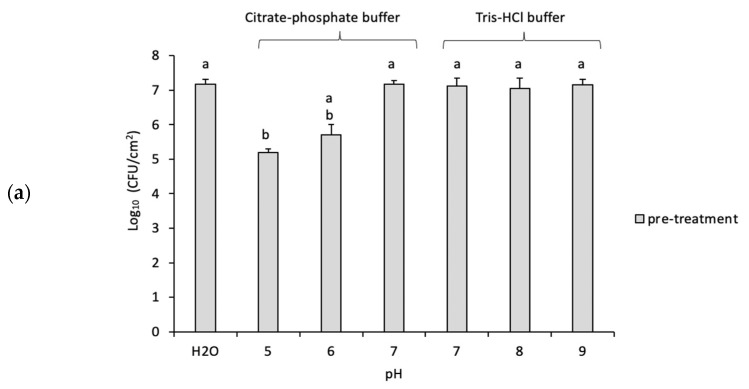

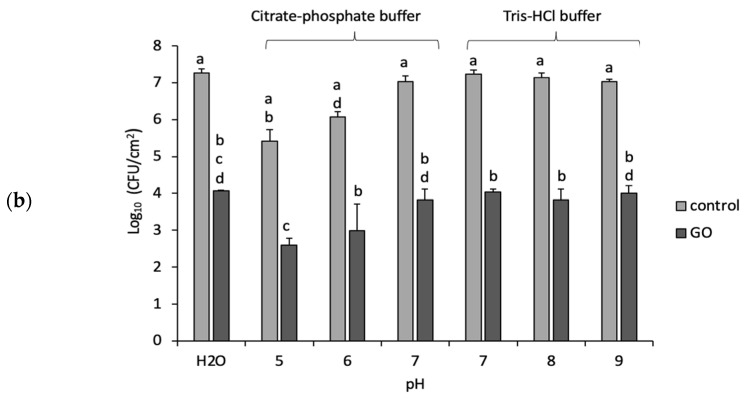
Impact of different constant pH values on *P. putida* biofilm (**a**) The viability of biofilm after exposure to different pH values (pre-treatment). (**b**) The viability of biofilm after the exposure to constant pH and incubation with either deionized water (control) or 85 mg/mL of GO for 24 h (post-treatment). Mean values with different letters are significantly different (*p* < 0.05). Similar letters are non-significantly different. The data were analyzed with one-way ANOVA.

The impact of pH on biofilms is demonstrated in Figure 2a,b which compares the treatment of biofilms with the different pH values, pre- and post-treatment with GO. Herein, we found a direct correlation between the cell viability and pH. For instance, the lower the pH value (5 and 6), the lower the viability of bacterial cells and vice versa until pH 7. The pre-treatment samples showed a slight but not significant reduction in CFU at low pH such as pH 5 (~1.5 log) and pH 6 (~1 log) after incubation for 3 h compared with the initial sample (Figure 2a). However, at neutral or higher pH (pH 7, 8 and 9), there was little or no effect on biofilm cell viability. The post-treatment biofilms incubated with 85 µg/mL GO showed a significant reduction in CFU (~3 log) at all pH ranges. Nevertheless, pH was not found to significantly influence the anti-biofilm activity of GO as shown in Table 3. At pH 5, the differences between pre-treatment and GO were (2.60 ± 0.29) and the differences between pre-treatment and GO were (3.35 ± 0.41) and (3.15 ± 0.35) at pHs 7 and 9, respectively. 

**Figure 3:**
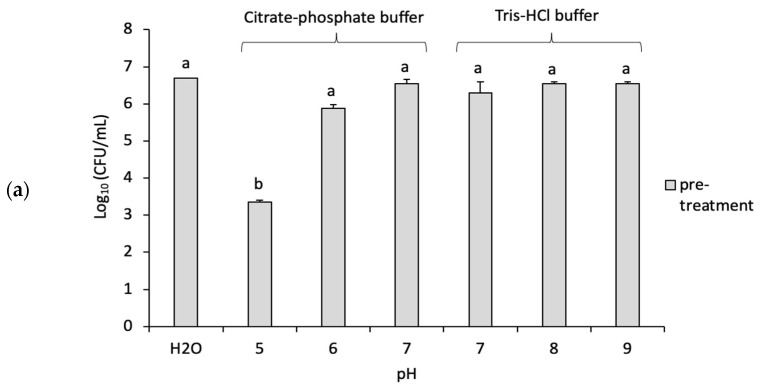

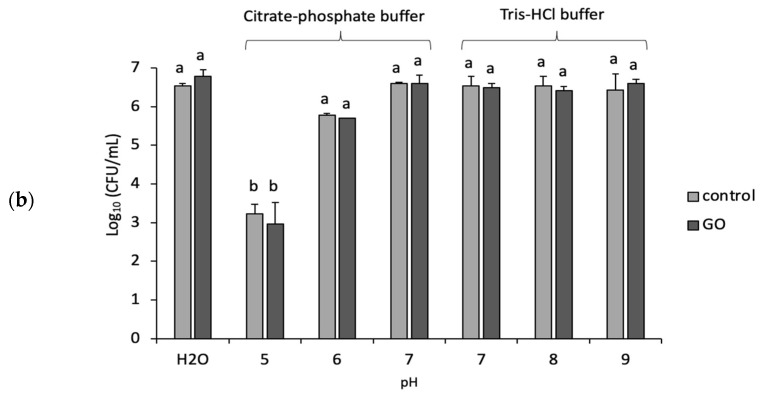
Impact of different pH values on *P. putida* planktonic cells (**a**) The viability of the pre-treatment samples of planktonic cells after exposure to different pHs (**b**) The viability of the control samples (deionized water) or the post-treatment samples (GO at 85 mg/mL) of planktonic cells after the exposure to a constant pH. Mean values with different letters are significantly different (*p* < 0.05). Similar letters are non-significantly different. The data were analyzed with one-way ANOVA.

The effect of pH stress and GO on planktonic cells is shown in Figure 3. Unlike biofilms, the CFUs of the pre-treated planktonic cells were significantly (*p* < 0.05) reduced (~3 log) at lower pH (pH 5) and with ~0.5 log at pH 6 compared with the initial ~6.5 log CFU/mL. This is potentially due to the protective nature of the EPS layer in the biofilm against the acidic environment. In addition, there was no significant difference in CFUs at the other pH values studied. There was also no significant difference in CFUs following the exposure of cells to 85 µg/mL GO compared to the pre- or post-treatment control samples at the same pH values, as shown in Figure 3.

### 3.4. Effect of Osmotic Stress

**Figure 4:**
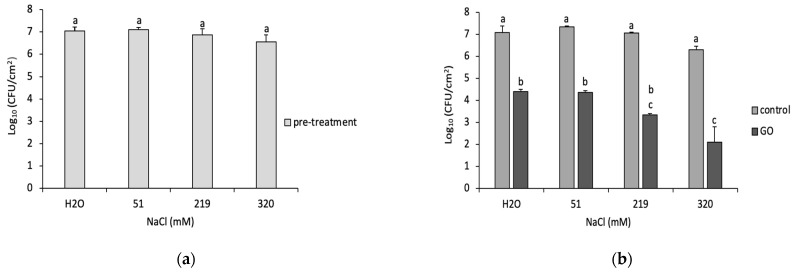
Impact of different osmotic pressure on *P. putida* biofilm cells (**a**) The viability of biofilm after the exposure to different NaCl concentration (pre-treatment). (**b**) The viability of biofilm after the exposure to constant NaCl concentrations (51, 219 and 320 mM) and incubation with either deionized water (control) or 85 mg/mL GO for 24 h (post-treatment). Mean values with different letters are significantly different (*p* < 0.05). Similar letters are non-significantly different. The data were analyzed with one-way ANOVA.

To study the effects of osmotic stress and GO on biofilms, the mature biofilm was incubated for 3 h at different NaCl concentrations (pre-treatment) and then further incubated for 24 h with either H_2_O (control) or 85 µg/mL GO (pre-treatment). The influence of osmotic stress on bacteria is shown in Figure 4. At concentrations of 51 mM and 219 mM NaCl, the cell viability remained similar to that of the control (in water), which was not exposed to the stress. However, at a higher concentration (320 mM NaCl), there was a slight reduction (~0.5 log) in CFU/cm^2^ as shown in Figure 4a.

In contrast, biofilms treated with GO after exposure to NaCl stress for 3 h (post-treatment) showed a significant (*p* < 0.05) decrease in CFUs for all NaCl concentrations. This reduction was significantly (*p* < 0.05) higher when the NaCl concentration increased. As shown in Figure 4b, the CFU/cm^2^ for biofilms exposed to GO at 219 mM was ~2.8 log and 1.6 log at 320 mM of NaCl. 

Further calculations were carried out to investigate the reduction between pre- and post-treatment as shown in Table 4. For example, the reductions in CFUs at 0 (H_2_O) and 51 mM NaCl were 2.64 ± 2.74 log and 2.74 ± 0.13 log, respectively. However, the CFUs decreased to 3.52 ± 0.22 for 219 mM and 4.45 ± 0.71 for 320 mM, when compared with the pre-treated samples.

**Figure 5:**
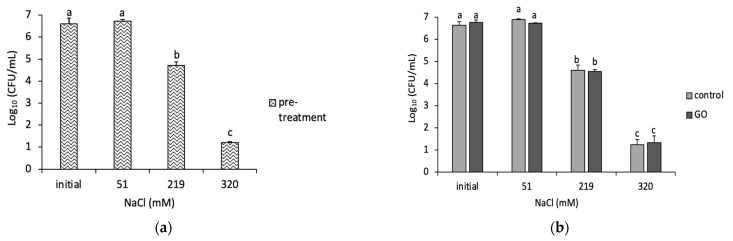
Impact of different osmotic pressure (using NaCl) on *P. putida* planktonic cells. (**a**) The viability of planktonic cells after exposure to various concentrations of NaCl (pre-treatment). (**b**) The viability of planktonic cells following 24 h exposure to constant NaCl concentrations (51, 219 and 320 mM) and incubation with deionized water (control) or 85 mg/mL GO. Mean values with different letters are significantly different (*p* < 0.05). Similar letters are non-significantly different. The data were analyzed with one-way ANOVA.

The impact of GO on planktonic cells at different concentrations of NaCl is shown in Figure 5. The CFUs of the pre-treated cells at high concentrations of NaCl showed a reduction of ~1.2 log for 219 mM and ~4.6 log for 320 mM. The controls in the post-treatment samples showed no differences when compared with their pre-treatment counterparts. Moreover, the samples exposed to GO showed no significant differences when compared to the controls in the post-treatment. However, when comparing the GO-exposed sample at 320 mM NaCl to the control at 0 mM NaCl, the number of CFUs was reduced from ~6.5 log to ~1.5 log. However, this high log reduction was solely due to the presence of high NaCl content (i.e., 320 mM) regardless of GO.

The differences between the planktonic cells and biofilms at the high NaCl concentrations are obvious. The cell survival decreased at 219 and 320 mM in planktonic cells. However, the survival of biofilm cells was not affected at the same concentrations. This could be due to the protective nature of EPS to cells.

**Figure 6:**
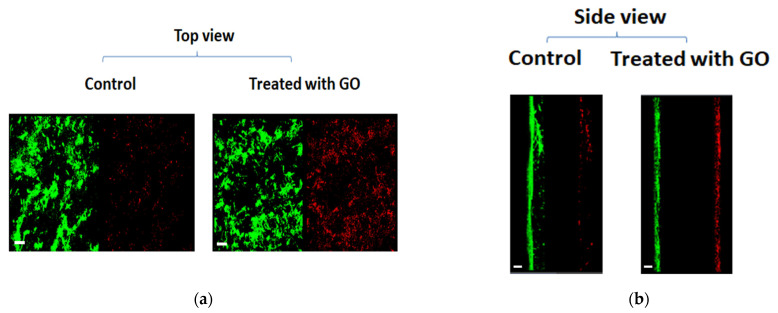
Confocal images of *P. putida* biofilm with or without GO (85 µg/mL) following 48 h incubation with 320 mM NaCl. The biofilms were stained with SYTO 9 (left, green) and PI (right, red). (**a**) Top view (**b**) side view. Scale bar: 20 µm.

Moreover, confocal microscopy was used to assess the membrane integrity of *P. putida* biofilm cells to assess the damage or the death of bacterial cells. Figure 6 shows the biofilms after treatment with 320 mM NaCl with and without GO exposure. The images from confocal microscopy showed an increase in PI staining after GO treatment compared to the control, indicating a decrease in membrane integrity in the presence of GO. These results confirm the CFU data (Figure 4b) that showed a reduction in cell viability following GO treatment compared to the control.

## 4. Discussion

### 4.1. Temperature

The aim of our study was to investigate the antibacterial activity of GO on planktonic and biofilm *P. putida* with or without pH, osmotic or temperature stresses. The effect of temperature on biofilms and planktonic cells incubated with GO was studied. Most *P. putida* biofilms form at 25 °C [13]. In the literature, 5 and 40 °C were used as the lowest and highest temperatures, respectively, to investigate the effects of temperature on *P. putida* biofilms [2,14,15,16,17], and based on that, those temperatures were chosen in our study. Moreover, Fallatah et al. (2019) studied the effects of two GO concentrations (8.5 and 85 μg/mL) on *P. putida* biofilms [11]. The lower concentration showed no antibacterial activity against biofilms while the higher concentration reduced the viability of biofilms by ~3-logs. Therefore, the concentration of 85 μg/mL GO was used in this study [11].

As expected, at all temperatures, the viability of *P. putida* biofilms was significantly (*p* < 0.05) reduced after exposure to GO compared to control. However, the temperature alone was not found to influence the viability of *P. putida* biofilm cells upon their in-parallel exposure to GO.

In this study, temperature was not shown to affect the viability of *P. putida* biofilm cells. Ricker and Nuxoll, (2017) studied the effect of antibiotics after heat shock (ranging from 37 to 80 °C for 1 to 30 min) on the viability of *P. aeruginosa* biofilms [4]. The viability of *P. aeruginosa* biofilms was reduced after shock treatment combined with antibiotics. The contradiction between our data and the literature may be due to the difference in the mechanism of killing that is induced by GO. However, further work needs to be carried out to confirm if this is true.

In addition, the lowest and highest temperatures (5 and 40 °C) tested in this study did not show any effects on planktonic cells. This observation is similar to a previous study by Karpouzas et al. [16]. However, in our study, the biofilms showed a slight reduction in the number of viable cells at 40 °C. This reduction was also observed in other studies such as that by Lin et al., who showed a reduction in the survival of *P. putida* biofilms at higher temperatures [6]. They demonstrated there is a reduction in bacterial growth at 35 °C, with some of the components of the exopolysaccharide (EPS) increasing such as carbohydrates, DNA and proteins. In biofilms, maintaining a healthy EPS is essential to prevent cells from dying at high temperatures. Furthermore, high temperatures may cause cell lysis and death in biofilms and planktonic cells. *P. aeruginosa* biofilms have also been shown to be more resistant to heat and antibiotic treatments compared to planktonic cells [4]. In our study, GO showed no effect on planktonic *P. putida* but was able to significantly reduce the viability of *P. putida* biofilm cells. 

### 4.2. pH Stresses

Due to the aggregation of GO at low pH, biofilms had to be pre-treated with different pHs before exposure to GO in water. This aggregation of GO was previously reported by Shih et al., who studied the behavior of GO at different pH values and found that at a low pH, GO aggregates [18]. This is due to the carboxyl groups in GO becoming extremely protonated, rendering it less hydrophilic. However, GO does not aggregate in water and shows a GO-water-GO sandwich-like structure that makes it stable instead of precipitating as confirmed by the potential of mean force (PMF) calculations. On the contrary, at high pHs, GO carboxyl groups become deprotonated, rendering the GO well dispersed in water to show a homogeneous dark-brown dispersion [6]. 

Our study highlights the strong impact of pH on *P. putida* biofilm and planktonic cells. The pre-treated samples, whether biofilms or planktonic, have been affected by low pH which reduced the viability of cells more than what is seen at higher pHs. Lee et al. studied the effect of pH range (4.0 to 10.0) on the EPS production in *P. putida* KT2440 using HCl or NaOH [19]. At higher pH, the production of polysaccharide and pellicle formation was enhanced while the latter was reduced in acidic buffers. A similar study found *P. putida* biofilm production being inhibited at low pH while the amount of EPS increased [6]. Thus, lowering the pH surrounding biofilms using citrate–phosphate buffer might be sufficient to suppress *P. putida* growth.

However, high pH did not show any statistically significant toxicity on the cells. These results agreed with van der Waal et al. who investigated the effect of alkaline buffers on mature *P. aeruginosa* biofilms using calcium hydroxide Ca(OH)_2_ at pH 12.1 [10]. The study showed that the alkaline broth did not reduce the number of bacterial cells. This was due not only to genetic adaption but also to the increase in EPS production which protected the cells in the alkaline environment [10,19]. However, this finding is contrary to previous studies which have suggested that high pH, similar to the ones used in our study, can exert a killing effect on planktonic forms of Gram-negative bacteria [20,21]. A study by Mendonca et al. treated two Gram-negative bacteria (*E. coli* and *Salmonella Enteritidis*) with NaOH buffers of pH 9.0–12.0 and found that these bacteria lost their viability at high pH values more than the Gram-positive bacteria (*Listeria monocytogenes*) [20]. This study suggested that the destruction of bacteria is more likely due to the disruption of the cytoplasmic membrane, which caused the internal contents to leak. This discrepancy between our study and some others could be due to species differences but also to the fact that biofilm cells are less susceptible to higher pH compared to planktonic cells. 

### 4.3. Osmotic Stress

Like pH, osmotic stresses were chosen according to the tolerance of cells to the chosen NaCl concentrations but without being totally destroyed [6]. The aggregation of GO in NaCl is showing in Figure S3. Previous studies have suggested that dispersed GO will aggregate in the presence of an electrolyte such as NaCl [22]. Electrolytes decrease significantly the electrostatic repulsion between the GO nanoparticles, which results in their destabilization by electrostatic double-layer attraction [22]. Wang et al. studied the behavior of different GO sizes in common inorganic salt electrolytes, such as NaCl, MgCl_2_ and AlCl_3_, at different concentrations. They found that the GO dispersions started to aggregate around 24 mM NaCl, but the dispersions remained stable at lower concentrations [23]. A similar phenomenon was found with other nanoparticles such as carbon nanotubes [24] and gold nanoparticles [25]. A study showed the effect of NaCl concentration on adsorption efficiency which increased with the increase in GO aggregation [26]. Thus, our study of osmotic stresses on bacteria and GO was completed following the pre-treatment of bacterial cells with NaCl. 

In our study, biofilms were slightly affected at higher concentrations of NaCl (Figure 4a). Interestingly, biofilms and planktonic cells were not affected at low concentrations of NaCl. However, at higher concentrations, the number of planktonic cells decreased compared with biofilms. Salts such as NaCl can alter the membrane fatty acid composition [3]. Moreover, the cells will shrink due to the osmotic pressure being higher outside the cell [27]. Thus, the number of planktonic cells decreased as the NaCl concentration increased. Lin et al. showed that EPS creates a more hydrated microenvironment around the cells, which, therefore, increases the adaptation of *P. putida* to osmotic stress without decreasing the growth rate [6]. However, the EPS production cannot protect the cells from plasmolysis caused by NaCl. Biofilms were convoluted (morphologically altered) under AFM, which indicates that NaCl exerted osmotic pressure on the cytoplasmic membrane even though the cells were surrounded by EPS. Moreover, Lin et al. found clear changes in the surface morphology of *P. putida* biofilms under different osmotic stresses with the cells’ volume decreasing slightly [6]. When the biofilms were exposed to 120 mM NaCl, the surface was extremely rough. Therefore, it is possible that at higher osmotic pressure, the bacterial membrane changes its activities due to increased membrane fluidity. This can make the cells in the biofilm more susceptible to damage from GO, as observed in our study, which highlighted a significant decrease in viability with GO at higher NaCl concentrations (Figure 4a). 

In addition, confocal microscopy images confirmed the CFU results highlighting cell death or damage at high concentration of NaCl. This suggests that the tolerance of biofilm to GO depends on the concentration of NaCl. The mechanism of destruction of bacterial cells by hyperosmotic stress has been reported earlier by others [28,29]. Microorganisms organize their intracellular concentration of solute by counteracting the migration of water from inside to outside the cells. In the case of high osmotic stress outside the cells, the toxicity depends on the capability of the bacteria cells to adapt to the changed osmotic stress as resources of solutes and energy outside cells during hyperosmotic stress changes. Microorganisms will also be affected and destroyed when solutes or energy resources are low [30]. The phenomenon of osmotic stress depends on the solute’s concentrations which cause the movement of solvents between the cell and its environment until the solute’s concentrations reach an equilibrium inside and outside the cells.

## 5. Conclusions

In conclusion, the effects of temperature, pH and osmotic stresses on biofilms followed by GO exposure were studied. GO exerted the same effect on cells at all temperatures. However, the combined exposure of low pH and GO affected biofilms. Moreover, the biofilms were affected by the combined exposure to GO with high osmosis. In general, planktonic cells were not affected by GO. As highlighted by Fallatah et al. who previously showed that the 48 h old biofilms were more sensitive than planktonic cells to GO, herein, we speculate that the gene expression or the secretion of molecules at this stage of biofilm formation may be responsible for this susceptibility to GO [11].

The results showed that the exposure to GO had no additional or synergistic antibacterial activity against biofilms and planktonic cells that have been subjected to temperature and pH stresses. Conversely, higher concentrations of NaCl reduced the population of biofilms. These findings suggest that *P. putida* can survive in a wide range of environmental stresses. Moreover, some of these stresses, such as osmotic stress, could also lead to biofilms becoming more susceptible to GO toxicity probably due to physiological changes in the cell membrane. To better understand how these stresses, alongside GO exposure, affect *P. putida*, it is important to study changes that occur to the bacteria at the molecular level, such as genes and proteins. Besides molecular effects, the higher toxicity of GO in the presence of osmotic stress against biofilm cells may also be related to changes in some biofilm specific features such as changes in EPS consistency. It is also important to study and investigate the bacterial cell membrane to understand the mechanism of GO toxicity. These findings can be interesting for other researchers investigating the toxicity of GO on bacteria.

## Figures and Tables

**Table 1:** Final volumes needed to achieve the acidic buffers.

pH	5.0	6.0	7.0
mL of sodium phosphate (0.2 M)	25.7	33.1	43.6
mL of citric acid (0.1 M)	24.3	16.9	6.5
deionized water (mL)	50.0	50.0	49.9
final volume (mL)	100.0	100.0	100.0

**Table 2:** Final volumes needed to achieve basic buffers after making 50 mL of Tris(hydroxymethyl)aminomethane (0.1 M).

pH	7.0	8.0	9.0
mL of HCl (0.1 M)	44.2	29.2	5.0
deionized water (mL)	155.8	170.8	195.0
final volume (mL)	200.0	200.0	200.0

**Table 3:** Differences in log_10_ CFU/cm^2^ viability between pre-treatment and GO; control and GO.

	H_2_O	5	6	7	7	8	9
(Pre-)-GO	3.10 ± 0.15	2.60 ± 0.29	2.71 ± 1.02	3.35 ± 0.41	3.08 ± 79	3.22 ± 0.61	3.15 ± 0.35
Cont-GO	3.36 ± 0.12	2.82 ± 0.13	3.07 ± 0.57	3.19 ± 0.03	3.02 ± 0.28	3.21 ± 0.46	3.32 ± 0.17

**Table 4:** Differences in log10 CFU/cm^2^ between (pre-treatment) GO and control GO.

	H_2_O	51 mM	219 mM	320 mM
(pre-)-GO	2.64 ± 0.06	2.74 ± 0.13	3.52 ± 0.22	4.45 ± 0.71
Cont-GO	2.69 ± 0.27	2.98 ± 0.04	3.52 ± 0.01	4.45 ± 0.38

## Data Availability

All the data presented here are available on request from the corresponding authors.

## Supplementary Materials

The following supporting information can be downloaded at: https://www.mdpi.com/article/10.3390/microorganisms11030609/s1, Figure S1: Flow chart illustrating the experimental steps in the study used to detect the viability of biofilm in pH ranges 5, 6, and 7; Figure S2: Photographs of GO incubated in low and high pH; and with 320mM NaCl for 0 and 24 h (**A**) Shows the GO in pH 5 and 9. After 24 h, GO aggregated at pH (**B**) A top photo of a 6-well plate showing the stability of GO in water and with 320 mM NaCl. After 24 h, GO aggregate following mixing with 320mM of NaCl. The black arrow indicates the aggregation.
