# Supplementary material for: Impact of Environmental Stresses on the Antibacterial Activity of Graphene Oxide (GO) Nanoparticles against P. putida Biofilms
Source: Microorganisms. 2023 Feb 28;11(3):609. doi: 10.3390/microorganisms11030609 (PMC10058726; doi:10.3390/microorganisms11030609)
Supplement: Supplementary file 1 [file microorganisms-11-00609-s001.zip › microorganisms-2213565-supplementary.pdf]

Article

Impact of Environmental Stresses on the Antibacterial Activity of Graphene Oxide (GO) Nanoparticles against *P. putida* Biofilms

Hussam Fallatah [1,2,3,*], Tim Overton [2], Hanene Ali-Boucetta [3,*] and Konstantinos Gkatzionis [4]

[1] Waste Management and Recycling Institute, King Abdulaziz City for Science and Technology (KACST), Riyadh 12354, Saudi Arabia
[2] School of Chemical, Engineering, University of Birmingham, Birmingham B15 2TT, UK
[3] Nanomedicine, Drug Delivery & Nanotoxicology (NDDN) Laboratory, School of Pharmacy, University of Birmingham, Birmingham B15 2TT, UK
[4] Department of Food Science and Nutrition, University of the Aegean, Metropolite Ioakeim 2, 81400 Myrina, Lemnos, Greece
* Correspondence: hfallatah@kacst.edu.sa (H.F.); h.aliboucetta@bham.ac.uk (H.A.-B.)

Supplementary Materials

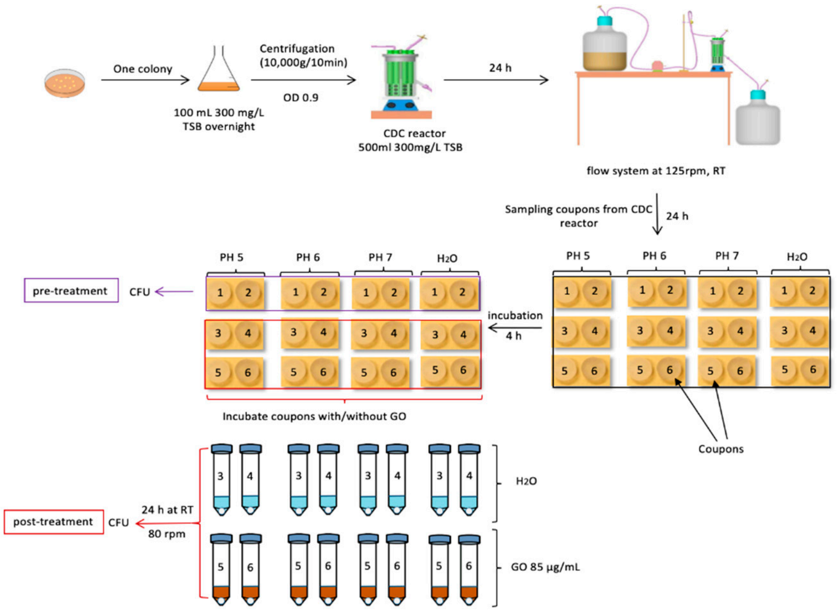

Figure S1 Flow chart illustrating the experimental steps in the study used to detect the viability of biofilm in pH ranges 5, 6, and 7

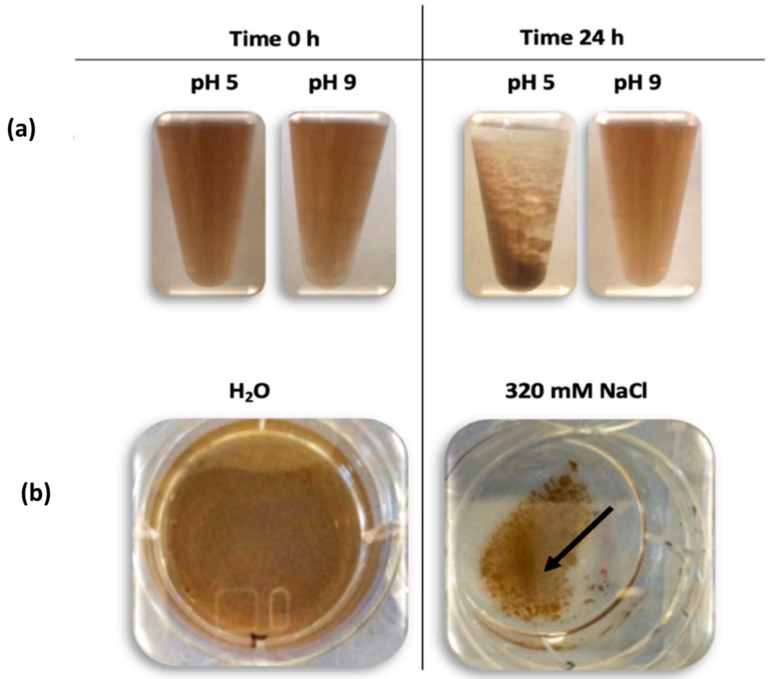

Figure S2 Photographs of GO incubated in low and high pH; and with 320mM NaCl for 0 and 24 h (**A**) Shows the GO in pH 5 and 9. After 24 h, GO aggregated at pH (**B**) A top photo of a 6-well plate showing the stability of GO in water and with 320 mM NaCl. After 24 h, GO aggregate following mixing with 320mM of NaCl. The black arrow indicates the aggregation.
